# Sparse Spatiotemporal Descriptor for Micro-Expression Recognition Using Enhanced Local Cube Binary Pattern

**DOI:** 10.3390/s20164437

**Published:** 2020-08-08

**Authors:** Shixin Cen, Yang Yu, Gang Yan, Ming Yu, Qing Yang

**Affiliations:** 1 School of Electronic and Information Engineering, Hebei University of Technology, Tianjin 300401, China; 201711901001@stu.hebut.edu.cn (S.C.); 201711901008@stu.hebut.edu.cn (Q.Y.); 2 School of Artificial Intelligence, Hebei University of Technology, Tianjin 300401, China; yuyang@hebut.edu.cn (Y.Y.); yangang@hebut.edu.cn (G.Y.); 3 Department of Electronic and Optical Engineering, Army Engineering University Shijiazhuang Campus, Shijiazhuang 050000, China

**Keywords:** micro-expression recognition, enhanced local cube binary pattern, multi-regional joint sparse learning, multi-kernel support vector machine

## Abstract

As a spontaneous facial expression, a micro-expression can reveal the psychological responses of human beings. Thus, micro-expression recognition can be widely studied and applied for its potentiality in clinical diagnosis, psychological research, and security. However, micro-expression recognition is a formidable challenge due to the short-lived time frame and low-intensity of the facial actions. In this paper, a sparse spatiotemporal descriptor for micro-expression recognition is developed by using the Enhanced Local Cube Binary Pattern (Enhanced LCBP). The proposed Enhanced LCBP is composed of three complementary binary features containing Spatial Difference Local Cube Binary Patterns (Spatial Difference LCBP), Temporal Direction Local Cube Binary Patterns (Temporal Direction LCBP), and Temporal Gradient Local Cube Binary Patterns (Temporal Gradient LCBP). With the application of Enhanced LCBP, it would no longer be a problem to provide binary features with spatiotemporal domain complementarity to capture subtle facial changes. In addition, due to the redundant information existing among the division grids, which affects the ability of descriptors to distinguish micro-expressions, the Multi-Regional Joint Sparse Learning is designed to perform feature selection for the division grids, thus paying more attention to the critical local regions. Finally, the Multi-kernel Support Vector Machine (SVM) is employed to fuse the selected features for the final classification. The proposed method exhibits great advantage and achieves promising results on four spontaneous micro-expression datasets. Through further observation of parameter evaluation and confusion matrix, the sufficiency and effectiveness of the proposed method are proved.

## 1. Introduction

As a spontaneous, low-intensity facial expression, micro-expression (ME) is a non-verbal way of expressing emotions, and it reveals the inner emotional state of human beings [1]. Lasting for only 1/3 to 1/25s, ME is usually an unconscious appearance of an emotional state, and can be detected even when a person tries to hide or suppress emotions. These involuntary expressions provide insight into the true feelings of a person. Due to these facts, MEs can provide valuable clues for emotional intelligence research, polygraph detection, political psychoanalysis, and clinical diagnosis of depression [2]. Thus, the study of MEs has recently attracted widespread attention at the forefront of psychology [3,4,5].

Since MEs are almost imperceptible to human beings, their subtlety and brevity impose significant challenges for observation with the naked eye. In this field, Ekman developed the first ME training tool (METT) [6]. After METT training, the performance of human subjects improved greatly. Even so, it was reported that the performance of human subjects in identifying MEs was only 47% on the post-test phase of METT [7]. It can be clearly seen that, although the study of MEs is an active and well-established research field in psychology, the research on ME recognition, especially in combination with computer vision, is still in its infancy and needs to be further developed. Furthermore, the automatic recognition of MEs has great significance in the mechanisms of computer vision, psychological research, and other practical applications. In consequence, exploring machine vision techniques for ME recognition has important research value and urgency.

The ME recognition research based on computer vision includes three steps: pre-processing, feature representation, and classification [8]. The development of powerful feature representations plays a crucial role in ME recognition, which is similar to other image classification tasks. Therefore, feature representation has been developed into one of the main focus points of recent research on ME recognition [9]. Currently, advances in computer vision-based image classification, such as machine learning and deep learning algorithms, provide unprecedented opportunities for automated ME recognition. In this context, many handcrafted techniques and deep learning methods have been proposed to predict MEs using different feature extraction methods, yielding valuable insights into the progression patterns of ME recognition [10,11,12,13,14,15,16,17,18,19,20,21,22,23,24,25,26,27,28]. The mainstream feature representation methods for ME recognition are mainly based on the optical flow [13,14,15,16,17], local binary patterns (LBP) [18,19,20,21,22,23], and deep learning techniques [24,25,26,27,28]. With their robustness to subtle facial changes, early studies of LBP and optical-flow-based handcrafted techniques have gradually become the benchmark methods for ME recognition tasks. LBP-based descriptors can obtain high-quality facial texture, which helps improve the representation ability of facial movements. The optical-flow-based studies also follow the idea of capturing facial details to describe motion information between adjacent frames, and many studies demonstrated their effectiveness for ME recognition tasks. Constrained by the sensitivity to the illumination, most of the optical-flow-based studies suffer from the interference of background noise, which limits their applicability. In recent years, the deep learning technology, with a rapid revival, has made it possible to avoid complicated handcrafted features design [28]. However, the expected performance is achieved by requiring a large number of training samples. Since it is difficult to induce spontaneous MEs and label them, the lack of large-scale benchmark datasets has considerably limited the performance of deep models. Therefore, due to the lack of large-scale datasets for the deep learning methods, mainstream ME recognition methods are still dominated by the traditional handcrafted techniques.

Based on reports, LBP, a typical handcrafted technique, is widely used in various pattern recognition tasks due to its outstanding advantages, such as computational efficiency, robustness to gray-scale changes, and rotation invariance. Similarly, as a three-dimensional (3D) extension of LBP, local binary patterns from three orthogonal planes (LBP-TOP) was first applied for the ME recognition task [18], which compensates for the shortcomings of LBP in time-domain features encoding. Although the spatiotemporal features show improvements in ME recognition performance compared with the traditional LBP features, the indirect acquisition of LBP-TOP features may introduce noise, which cannot be ignored. Moreover, different variant studies based on LBP-TOP only reveal information related to the three orthogonal planes, which leads to the loss of some hidden clues [21]. Due to these facts, the representational capabilities in the time-domain is weakened to some extent by adopting the coding patterns of three orthogonal planes. Besides, the handcrafted techniques can be designed to identify discriminative information in specific tasks, which allows different variants of LBP to have different coding concerns. As a result of using only one type of binary feature, the representation ability and robustness are inefficient or insufficient. On such occasions, the integration of multiple complementary descriptors (e.g., the complementary spatial and temporal descriptors), while bringing a high redundancy of data and thus compromising the quality of the representation, can compensate for the limitations of the spatiotemporal representation in a single descriptor. In fact, the high-dimensional problems with redundant information pose a severe challenge in various fields. Further, as the LBP-based technique introduces division grids to capture local information, this leads to a difference in the contribution of different local regions to the recognition task. In response to this issue, the adoption of a group sparse regularizer provides cues and references for conducting feature selection and calculating the contribution of different regions. Moreover, the impact of different sparse strategies on recognition performance is particularly important. In such a context, choosing the appropriate sparse strategy and combining it with different types of binary encoding patterns would be a better choice for ME recognition tasks. Moreover, due to the lack of flexibility, a single kernel function may limit the performance of the task. Therefore, the representations mapped by the Multi-kernel Support Vector Machine (SVM) can be more accurately and reasonably expressed in the new combination kernel space for final classification, which makes the results of the ME recognition method more convincing.

**Figure 1:**
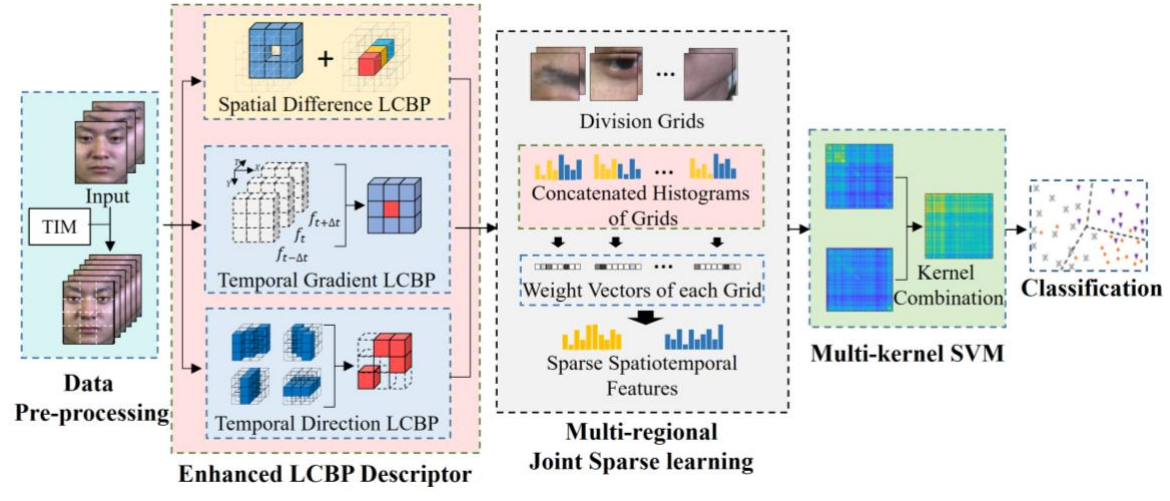
The framework of the proposed method, which mainly includes the Enhanced Local Cube Binary Pattern (LCBP) Descriptor, Multi-Regional Joint Sparse Learning, and Multi-kernel Support Vector Machine (SVM).

In this paper, a sparse spatiotemporal descriptor for ME recognition using Enhanced Local Cube Binary Pattern (Enhanced LCBP) is developed to classify MEs. The framework of the proposed method is shown in Figure 1. Our method emphasizes the integration of three complementary descriptors in the spatial and temporal domains, so as to improve the high distinctiveness and good robustness of the spatiotemporal representation. In response to the importance of spatiotemporal information for the ME recognition, the proposed complementary binary feature descriptors include Spatial Difference Local Cube Binary Pattern (Spatial Difference LCBP), Temporal Direction Local Cube Binary Pattern (Temporal Direction LCBP), and Temporal Gradient Local Cube Binary Pattern (Temporal Gradient LCBP). These descriptors are designed to capture spatial differences, temporal direction, and temporal intensity changes, respectively. The Enhanced LCBP not only takes full advantage of the LBP algorithm but also integrates more complementary feature extraction capability in the spatiotemporal domain. In addition, to direct the model to focus more on important local regions, Multi-Regional Joint Sparse Learning is designed to measure the contribution of different regions to distinguish micro-expressions, which captures the intrinsic connections among local regions. It not only reduces redundant information by using the sparse penalty term, but also employs an extra penalty term for constraining the distance between different classes in the feature space, thus further improving the discriminability of sparse representations. Furthermore, by mapping the sparse representations through a Multi-kernel SVM, it can be combined with the mapping capabilities of each kernel space, ultimately allowing for a more accurate and reasonable expression of the data in the synthetic kernel space. Finally, we performed comparative and quantitative experiments on the four benchmark datasets to study the effectiveness of our method.

The structure of this paper is as follows: Section 2 reviews the related work of mainstream ME recognition methods and presents the contribution of this paper. The proposed method is presented in Section 3. The experimental results and comparative analysis is discussed in Section 4. Section 5 provides the conclusion.

## 2. Related Work

State-of-the-art ME recognition methods can be broadly classified into three categories: optical flow, LBP descriptors, and deep learning algorithms. The related work of these three methods is briefly reviewed in this section.

The purpose of optical-flow estimation is to observe the facial motion information between adjacent frames. The optical-flow-based techniques calculate the pixel changes in the time domain and the correlation between adjacent frames to find the correspondence of facial action between the previous frame and the current frame. To answer the challenges of short duration and low intensity of MEs, Liu et al. [13] proposed a simple yet effective Main Directional Mean Optical-flow (MDMO) feature for ME recognition. MDMO is a normalized statistical function based on regions of interest (ROIs), which is divided based on the definition of action units. It divides the main optical flow into amplitude and direction according to the characteristics of the main optical flow in each region of interest. To extract subtle face displacements, Xu et al. [14] proposed a method to describe the movement of MEs at different granularities, called Facial Dynamics Map (FDM). They used optical flow estimation to pixel-align the ME sequences. They also developed an optimal iterative strategy to calculate the main optical flow direction of each cuboid, which is obtained by dividing each expression sequence according to the selected granularity. This is done in a purposeful way to characterize facial movements containing time-domain information to predict MEs. Further, in response to the problem that MDMO lost the underlying manifold structure inherent in the feature space, Liu et al. [15] proposed a Sparse MDMO feature that uses a new distance metric method to reveal the underlying manifold structure effectively. This improvement makes full use of the sparseness of the sample points in the MDMO feature space, which is obtained by adding new metrics to the classic Graph Regularized Sparse Coding scheme. Due to optical flow estimation that can capture dense temporal information, some researchers advocated using the key frames, or fuzzy histograms, to reduce the impact of redundant information on ME recognition. Specifically, Liong et al. [16] proposed a Bi-Weighted Oriented Optical Flow (Bi-WOOF) to encode essential expressiveness of the apex frame. Happy and Routray [17] proposed a Fuzzy Histogram of Optical Flow Orientations (FHOFO) method, which constructs an angle histogram from the direction of the optical flow vector using histogram fuzzification.

Taken together, the optical-flow field can effectively convey temporal micro-level variations in MEs. Since OP-based features can describe the location and geometry of refined facial landmarks, they require a more precise facial alignment process. This brings some complexity to the ME recognition process and the current models. Meanwhile, its sensitivity to illumination changes makes it highly susceptible to background noise, which causes the OP-based method to amplify local facial details while also amplifying redundant information.

Since the spatiotemporal features play an important role in identifying ME, these LBP-based studies on ME recognition have gradually developed towards improvement in encoding time-domain representations. Earlier work explored extending different coding patterns to three orthogonal planes to develop descriptors that are robust to time-domain changes, including LBP-TOP [18], Centralized Binary Pattern from three orthogonal planes (CBP-TOP) [19], etc. In particular, Pfister et al. [18] extended LBP to three orthogonal planes (LBP-TOP) to identify MEs. The LBP-TOP inherited the excellent computing efficiency of the LBP, which can effectively extract texture features in the spatiotemporal domain. Since then, the LBP-TOP has been used as a classic algorithm to provide the basis and verification benchmark for follow-up studies. Wang et al. [20] proposed a Local Binary Pattern with Six Intersection Points (LBP-SIP), which contains four points in the airspace and the center points of two adjacent frames. Guo et al. [19] proposed to extend the Centralized Binary Pattern (CBP) features in three orthogonal planes to CBP-TOP descriptors, thereby obtaining lower-dimensional and more enriched representations. Meanwhile, due to the problem of losing information by encoding only in the three orthogonal planes, we extended the LBP encoding to the cube space and proposed Local Cube Binary Patterns (LCBP) for ME recognition [21]. LCBP concatenated the feature histograms of direction, amplitude, and central difference to obtain spatiotemporal features, which reduces the feature dimension while preserving the spatiotemporal information. In addition to calculating the sign of pixel differences, Huang et al. [22] proposed a Spatiotemporal Local Binary Pattern with an Integral Projection (STLBP-IP), which is an integral projection method based on differential images. It obtains the horizontal and vertical projections while retaining the shape attributes of the face image, which enriches the time domain representation but loses some spatial details. To address this issue, they further proposed a Discriminative Spatiotemporal Local Binary Pattern with Revisited Integral Projection (DISTLBP-RIP) by incorporating the shape attribute into the spatiotemporal representations [23].

The LBP technique is more effective than the optical flow technique and therefore has a broader application. The coding patterns of existing methods are based on capturing time-domain information in three orthogonal planes. For these reasons, the motion cues in other directions, and the temporal correlation between adjacent frames are ignored to some extent. Although the LBP-based approach provides a refined description of the local texture, the relationship between different local regions and the relationship between the same local locations in different temporal sequences can still not be disregarded. Additionally, the higher feature dimensions and the redundancy of representations also constrain the model’s recognition performance. Based on the above discussion, it is not surprising to see that research on ME recognition based on LBP technology still has great promise. In particular, an effective combination of multiple coding patterns and correlation modeling between descriptors will make the results of the ME recognition approach more compelling.

In recent years, with its advantages of avoiding manual function design, the success of deep learning in a wide range of applications has been witnessed. Many researchers have tried to apply deep learning techniques to the field of ME recognition. Khor et al. [24] proposed an Enriched Long-term Recurrent Convolutional Network to predicted MEs. The network contains two variants of an Enriched LRCN model, including the channel-wise stacking of input data for spatial enrichment and the feature-wise stacking of features for temporal enrichment. However, due to the limited training data of the ME dataset, early attempts based on deep learning are difficult to achieve competitive performance. In order to leverage the limited data, Wang et al. [25] proposed a Transferring Long-term Convolutional Neural Network (TLCNN) to identify MEs. TLCNN used two-step transfer learning to solve the issue: (1) transfer from expression data, and (2) transfer from a single frame of ME video clips. Li et al. [26] developed a 3D-CNN model to extract high-level features, which uses optical flow images and gray-scale images as input data, thereby alleviating the issue of limited training samples. In addition, some studies mainly focus on the improvement of deep learning models to achieve better performance. Yang et al. [27] proposed a cascade structure of three VGG-NETs and a Long Short-Term Memory (LSTM) network LSTM, and integrated three different attention models in the spatial-domain. Song et al. [28] developed a Three-stream Convolutional Neural Network (TSCNN) by encoding the discriminative representations in three key frames. TSCNN consisted of a dynamic-temporal stream, static-spatial stream, and local-spatial stream module, which are used to capture the characteristics of temporal, entire facial region, and local facial region, respectively.

Indeed, the application of deep learning has provided an important boost to the study of ME recognition. Although deep learning models have been applied in studies of ME recognition with good results in some studies, the limited sample size still constrains the maximization of its potential. According to some reports, the main problem for researchers with deep learning models is in dealing with limited training data. Moreover, due to the trigger mechanism and spontaneity of MEs, it is extremely complex to construct large datasets for ME recognition tasks by deliberately controlling the subtle movements of facial muscles.

In accordance with the above discussion, the main idea of our solution is adequately extending the advantages of binary patterns to the time-domain for capturing rich facial movements, which is the key to ME recognition algorithms. For this purpose, three important issues need to be considered. To begin with, a suitable binary feature descriptor needs to be developed for the ME recognition task. Maintaining this binary encoding pattern with its theoretical simplicity, high computational efficiency, and excellent spatial texture representation capabilities is important. Unlike the traditional LBP-TOP, simply extending to the three orthogonal planes cannot capture the expected spatiotemporal variation. Due to the demand for spatiotemporal information for the ME recognition, we developed spatial and temporal descriptors emphasizing the complementary properties of the temporal and spatial domains. The proposed Enhanced LCBP integrates spatial differences, temporal directions, and temporal intensity changes in local regions, which aims to provide more evidence of facial action to predict MEs.

Second, Enhanced LCBP requires capturing local representations by division grids. Within this context, it is necessary to consider not only the relationships between the local regions triggered by MEs, but also the redundancy of information caused by the fusion of multiple descriptors. For this purpose, to enhance the sparsity and discriminability of the representation, the Multi-Regional Joint Sparse Learning is proposed to implement feature selection between different grids. Specifically, to prevent over-fitting caused by the high-dimensional vector, the $\ell_{2,1\;}$-norm penalty term is fused in the group sparse regularizer so that the different groups have similar sparse patterns. At the same time, interclass distance constraint is introduced to enhance the discriminative power of the sparse representations in feature space.

Third, the existing literature [29] reports that the kernel method is an effective way to solve the problem of nonlinear model analysis. However, for ME data, the classifier consisting of a single kernel function does not meet the practical needs of the sample data with uneven distribution of emotion categories and inflexible feature mapping capabilities. Therefore, it is a better choice to combine the different sparse representations separately through a multi-kernel SVM to obtain an optimal result.

To summarize, the contribution of this paper is presented as follows:

(1) In order to achieve our goal of fully exploiting the implied spatiotemporal information, an Enhanced LCBP binary descriptor is proposed that encodes subtle changes from different aspects, including spatial differences, temporal directions, and temporal intensity changes. Our research focuses on extracting the complementary features by integrating the spatial and temporal descriptors, thereby obtaining richer temporal variations that are robust to subtle facial action. With the help of descriptors, it is possible to provide binary representations containing more detail changes in the spatiotemporal domain, thus the discriminatory information implicit in the image sequence could be revealed;
(2) Second, to alleviate the interference of high-dimensional vectors on the learning algorithm, the Multi-Regional Joint Sparse Learning is designed to conduct feature selection, and thereby the discriminatory power of the representation is enhanced. Moreover, the proposed feature selection model can effectively model the intrinsic correlation among local regions, which can be utilized to focus the attention on important local regions, thus clearly describing the local spatial changes triggered by facial MEs;
(3) Sparse representations of spatial and temporal domains are mapped to different kernel spaces, aiming to exploit the different mapping capabilities of the individual kernel, which are further integrated into the synthetic kernel space. This takes full advantage of the more accurate and powerful classification capabilities of the multi-kernel SVM, especially for the fusion of multiple descriptors in our model, whose benefits are clear.

## 3. Proposed Method

As shown in Figure 1, the proposed method consists of three main parts: Enhanced LCBP, Multi-Regional Joint Sparse Learning, and multi-kernel SVM. In this section, the spatial and temporal descriptors of the proposed Enhanced LCBP is first presented. The other two parts are then explained in terms of their respective functions and how they help the model to improve recognition accuracy.

### 3.1. Enhanced Local Cube Binary Pattern (Enhanced LCBP)

**Figure 2:**
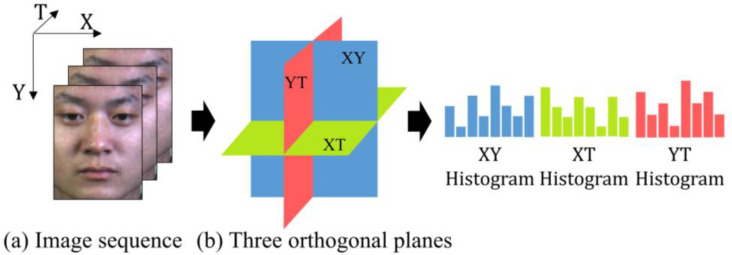
The feature extraction based on three orthogonal planes. (**a**) Three orthogonal directions of the image sequence, (**b**) The illustration of the three orthogonal planes.

The image feature descriptor, a fundamental but crucial technique for pattern recognition research, has the core design requirements of robustness and differentiation. Especially for challenging recognition tasks, developing image descriptors with more applicable features for different tasks is an effective way to improve the method overall. For challenging tasks like ME recognition, the effectiveness of the method can be enhanced by developing image descriptors with more adaptability for different tasks. As an excellent demonstration, LBP-TOP extends the encoding coverage to the three orthogonal planes in response to the loss of time-domain information in LBP, thus it can become one of the mainstream descriptors for ME recognition. To be specific, LBP considers capturing spatial textures, using the relationship between the center pixel and neighboring pixels. The difference between neighboring and center pixels is coded as 1 when it is non-negative, and 0 otherwise. Further, LBP-TOP extends this center-neighbor relationship to the three orthogonal planes. As shown in Figure 2a, X, Y, and T represent the three orthogonal directions in the three-dimensional coordinate system, where X, Y represents the spatial domain direction, and T represents the time domain direction. The three orthogonal planes shown in Figure 2b are the XY plane, XT plane, and YT plane. Within the three planes, the difference between the respective central pixels and the neighboring pixels is used to calculate the histogram, and the spatiotemporal features are eventually cascaded together to obtain.

Despite the fact that LBP-TOP integrates clues that exist in both the time-domain and spatial-domain, it has some limitations. Facial spatial features are the basis of emotional state analysis. The LBP-based methods all employ texture analysis of the XY plane to obtain facial spatial representations. However, for video image sequences, the correlation in spatial-domain is closely related to temporal domain variation. Specifically, due to the continuous spatial difference between two adjacent frames in the image sequence, this leads to a spatial coherence between adjacent frames that correlates with temporal variation, and the temporal facial action can be represented by spatial differences in binary features. This implies that the spatial difference not only expresses the relationship of spatial texture, but also reveals variation at the temporal level. In this context, the more detailed descriptions of spatial features and spatial differences can provide powerful clues to the ME recognition task. However, this correlation of local spatial differences between adjacent frames is easily disregarded. In other words, the missing consideration of the variation between the local spatial regions at different moments leads to an incomplete representation of the spatial coherence between adjacent frames. Simply focusing on spatial texture is not effective in capturing spatial differences. Therefore, the spatial difference needs to be further supplemented to the binary features.

According to reports [21], the spatiotemporal variation in facial movements provides strong evidence for analyzing the emotional state of MEs, which are conveyed by the changes in pixel brightness of local image sequences. Clearly, LBP-TOP captures the key to predicting MEs by converting three-dimensional temporal changes into two-dimensional temporal planes, thereby effectively capturing time-domain characteristics using spatially encoded patterns. This ingenious solution has been applied by many mainstream LBP-based methods to capture time-domain information. The cost of this solution, however, is that the temporal information only takes into account the pixel brightness changes in the two orthogonal planes (XT and YT). This inadequate sampling range limits the feature extraction capabilities of the model.

**Figure 3:**
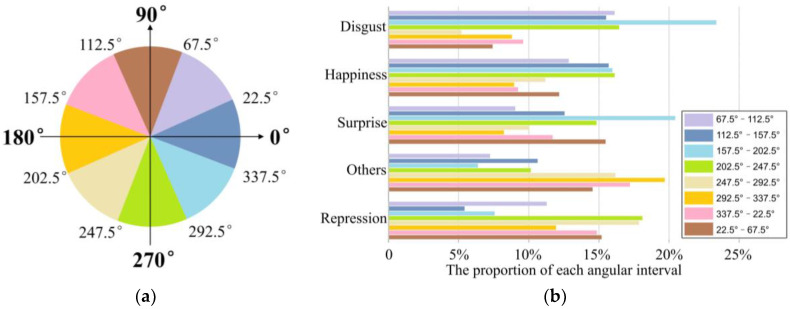
The statistical information on the temporal motion direction of different samples, which belong to the five major classes in Chinese Academy of Sciences Micro-expression2 (CASME-II). The statistics for temporal motion direction show a wide variation in the distribution of different samples, which leads to the fact that the sampling range of the temporal direction should be cover all directions. (**a**) The pie graph illustrates the division of eight angular intervals. Eight angular intervals contain the pixel moving direction in the XT, YT plane (i.e., XT:337.5°–22.5° and 157.5°–202.5°, YT:67.5°–112.5° and 247.5°–292.5°) and the non-orthogonal plane region (i.e., the other regions except in the XT and YT planes). (**b**) The directional statistical of pixel motion in eight angular intervals.

Moreover, while feature encoding in the three orthogonal planes does compensate for the loss of time-domain information, the problem of missing time-domain information remains. This is mainly reflected in the fact that the coding range is confined to three orthogonal plane areas. For the different feature encoding methods, the main purpose of encoding is to measure the facial action trend of the central pixels in the local region, which includes the temporal directions and the temporal intensity changes. Moreover, all of this spatiotemporal information, which manifests as changes in pixel brightness, is triggered by facial action. For the direction of facial action, our previous research of ME recognition demonstrates that facial actions are not limited to the temporal orthogonal planes [21]. Herein, we further use the ideas employed in our previous research to statistically distribute the temporal directions of the local facial action for different expression categories. Specifically, the Large Displacement Optical Flow [30] is used to extract the optical flow of each pixel moving between inter-frame, thereby obtaining the two-dimensional motion components $V_{x}$,$\;V_{y}$ of the pixel I(x,y,t) in the x and y directions. Then, the temporal directions of the pixels can be calculated as follows:

(1) $$H_{x,y}=\tan^{-1}\frac{V_{x}}{V_{y}}$$

where, $H_{x,y}$ denotes the angle at which the pixel I(x,y,t) moves to the next frame. Statistical information on pixel motion is calculated based on the moving angles of all pixels in the image sequence. Figure 3a exhibits the division of the pixel motion angular intervals that contain the orientation of the temporal motion in the XT, YT plane, and the non-orthogonal plane region. Figure 3b presents the proportion of the pixel motion direction appearing in the eight intervals for five major classes in Chinese Academy of Sciences Micro-expression2 (CASME-II). The statistical results show that the distribution of moving direction is different for the five expression categories. Considering that the capture of these temporal directions can provide more facial action clues for feature descriptors, the description of moving directions also needs to be fused into the spatiotemporal feature encoding.

Similar to the representation of the temporal directions, the temporal intensity changes in local regions are also the key to ME recognition. With respect to the facial action, the temporal intensity changes reveal the dominant information of the facial action trend. Therefore, the intensity changes in local regions are also important to supplement the spatiotemporal information of binary features.

Based on the above discussion, complementary descriptors need to be applied to capture different spatiotemporal features. This is the main distinction between handcrafted techniques and deep learning, and it is for this reason that there are binary features allowing the customization of encoding schemes for different tasks. Meanwhile, it has been reported that the encoding in cube space is more conducive to capturing valid ME information than the three-dimensional orthogonal plane, since the latter may introduce noise from features acquired indirectly [21]. Therefore, the proposed method also adopts cubic coding, but is different from LCBP. Actually, our method jointly encodes the spatial and temporal variations from the cube to construct complementary spatiotemporal feature descriptor. Specifically, the proposed Enhanced LCBP encodes the spatial difference, temporal direction, and temporal gradient between adjacent frames in the cube space, and these three binary feature descriptors are named as Spatial Difference LCBP, Temporal Direction LCBP, and Temporal Gradient LCBP, respectively. This not only inherits the advantages of LCBP for maintaining anti-noise performance and extending the sampling range, but it also exploits the spatial coherence associated with time variation and co-extraction of motion features with the encoding from multiple planes. Compared with LCBP, it sufficiently extends the application of pixel relationships within the cube to capture facial subtle changes in both the spatial and temporal domains.

#### 3.1.1. Spatial Difference Local Cube Binary Pattern (Spatial Difference LCBP)

**Figure 4:**
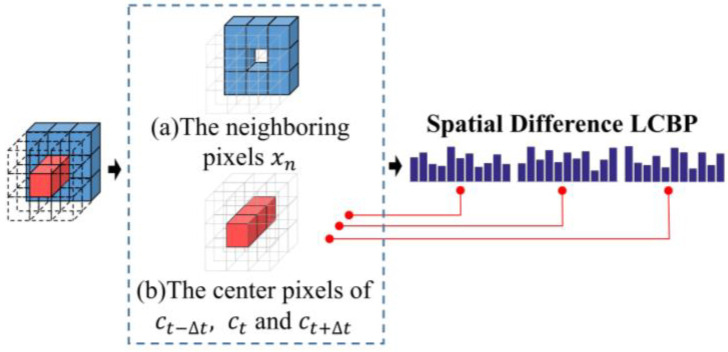
The encoding process of Spatial Difference LCBP. (**a**) The sampling range of the neighboring pixels in the cube space. (**b**) The sampling range of the center pixels in the cube space.

According to the above discussion, the spatial difference between adjacent frames within the same local area, i.e., within the cube space, is often an easily ignored problem. To supply spatial differences between adjacent frames, the Spatial Difference LCBP is proposed to enrich the spatial representation of binary features. Since the different binary patterns are essentially encoded around the relationship of the central pixel to the neighboring pixels, this eliminates the problem of light variation to some extent while gaining the advantage of better computational efficiency and gray-scale invariance. Along these lines, we extend this idea to encoding spatial difference, hoping to enrich feature representations by focusing on the variation in the relationship between different central pixels and neighboring pixels. Unlike LBP, the proposed binary pattern measures the relationship between different central pixels and the fixed neighboring pixels. Figure 4a illustrates a cube space of 3 × 3 pixel points at the same location from three adjacent frames. The blue positions inside the cube shown in Figure 4b indicate the neighboring pixels involved in the coding. The red position indicates the central pixel of the different frames involved in encoding. The transparent position indicates pixels that are not involved in the encoding.

Specifically, the three central pixels of the cube (red positions) calculate the difference with the neighboring pixels of the fixed frame (blue positions), respectively. The Spatial Difference LCBP code for each center pixel can be defined as:

(2) $$Spatial\;Difference\;LCBP\left(c_{i}\right)=\sum_{n=1}^{8}s\left(x_{n}-c_{i}\right)2^{n-1},\;s\left(x\right)=\{\begin{array}{c} 1\;x\geq 0\\ 0\;x<0 \end{array},$$

where, $x_{n}$ denotes the eight neighboring pixels of the frame $f_{t+\Delta t}$ in the cube space, and $c_{i}$ denotes the central pixels of the three frames in the cube, including $c_{t-\Delta t},\;c_{t}$ and $c_{t+\Delta t}$ respectively. *s*(·) is the sign function, which is used to calculate the binary codes. 

As shown in Figure 4, firstly, the Spatial Difference LCBP encodes the spatial texture of the third frame in the cube, by comparing the difference between the center pixel $c_{t+\Delta t}$ and the neighboring pixels $x_{n}$. Secondly, the Spatial Difference LCBP encodes the difference between the remaining two center pixels $c_{t}$ and $c_{t-\Delta t}$ and the neighboring pixels $x_{n}$. This allows the spatial feature relationships of adjacent frames to be quantified while the subtle variation signals of different spatial centers are encoded.

The histograms of the difference among the three center pixels and the neighboring pixels are calculated separately, and finally cascaded into a complete Spatial Difference LCBP feature, which contains the spatial central differences associated with the temporal variation. For this purpose, the trend of the central pixels within the cube is emphasized, and more evidence of facial movement is added to the spatial features.

#### 3.1.2. Temporal Direction Local Cube Binary Pattern (Temporal Direction LCBP)

Discussion based on the statistical diagram of the motion direction indicates the importance of temporal direction in facial action, which is the purpose of the proposed Temporal Direction LCBP.

**Figure 5:**
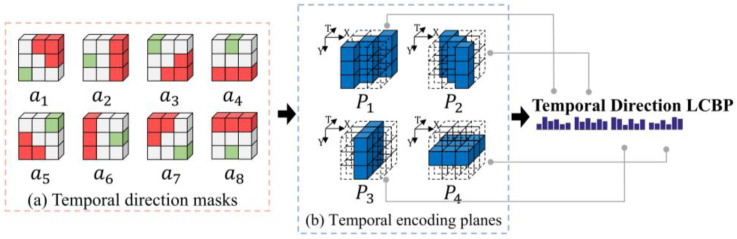
The encoding process of Temporal Direction LCBP. (**a**) The diagram of eight direction masks in the sampling plane. (**b**) The sampling range of the temporal planes in the cube space.

In our previous research [21], the compensation for the loss of temporal directions was proposed using the eight angles of the cube. Moreover, the experimental results also demonstrate the validity of our proposed method. With further research on motion directions, it is found that although the effect on feature dimension reduction is evident when processing the main direction of the cube using eight convolutional templates, this pattern only focuses on one out of the eight orientation angles. For the temporal directions of the facial action in the XT, YT planes, especially in the four orientation angles of the XT, YT plane (e.g., a1, a3, a5, a7 in Figure 5a), it is also necessary to be considered. This is due to the fact that the distribution of temporal directions varies significantly from sample to sample, and the encoding pattern should cover all existing directions. Meanwhile, the sampling sizes can affect the representational ability of descriptors. The temporal direction in cubes can be further explored by extending the sampling range to more time planes. 

Thus, as an improvement of LCBP, the proposed Temporal Direction LCBP exploits the directional response values in the multiple temporal planes to provide more evidence of facial actions, complementing the direction encoding in the XT and YT planes that the LCBP ignores. As shown in Figure 5, the blue regions $P_{1}$–$P_{4}$ presented in Figure 5b indicate the different temporal planes. The purpose of extending to multiple planes is to enhance the representation of the temporal direction, i.e., using motion information from the four planes to jointly represent the direction of motion at the center of the cube. Meanwhile, the overall sampling range is constrained to cover all pixels of the cube, making full use of the information provided by all pixels from the cube.

To be specific, the $a_{1}$–$a_{8}$ shown in Figure 5a are the eight directional masks. The red region of the directional mask is assigned as 1, the green region is assigned as −3, and the other regions are assigned as 0. The response values $D_{i}$ of the different directions are obtained by convolution of the eight directional masks $a_{1}$–$a_{8}$ with different temporal planes $P_{1}$–$P_{4}$:

(3) $$D_{i}\left(P_{j}\right)=a_{i}*P_{j},$$

where $P_{j}$ denotes the different time-domain planes in the cube, $a_{i}$ denotes the eight directional masks, and i=1,2,…,8, and ∗ denotes the convolutional operation. By calculating the maximum and minimum of the response values $D_{i}$ in the different planes, the directional code Temporal Direction LCBP can be obtained by the following equation:

(4) $$Temporal\;Direction\;LCBP\left(P_{j}\right)=2^{3}\times maxIndex\left(P_{j}\right)+minIndex\left(P_{j}\right),$$

In the above equation, $P_{j}$ denotes the different temporal planes in the cube space, and $maxIndex\left(P_{j}\right)$ and $minIndex\left(P_{j}\right)$ denote indexes of the maximum and minimum directional response values for each plane, respectively. Moreover,

(5) $$maxIndex\left(P_{j}\right)=arg\;\underset{i}{\max}\left\{D_{i+1}\left(P_{j}\right)|0\leq i\leq 7\right\},$$

(6) $$minIndex\left(P_{j}\right)=arg\;\underset{i}{\min}\left\{D_{i+1}\left(P_{j}\right)|0\leq i\leq 7\right\},$$

The four Temporal Direction LCBP codes extracted from the $P_{1}–P_{4}$ planes are used to calculate the temporal direction histograms for each plane. The four histograms are further cascaded to obtain the complete Temporal Direction LCBP feature. The combined efforts of the multiple temporal directions can provide more sufficient evidence to describe the motion of central pixels, complementing more temporal facial changes than the single direction of the LCBP.

#### 3.1.3. Temporal Gradient Local Cube Binary Pattern (Temporal Gradient LCBP)

In addition to the temporal direction and spatial differences, the intensity changes of the central pixels are also critical to describe facial action. An efficient way to represent the temporal intensity changes is to use the gradient information of the central pixels in the time domain direction. This is because the gradient information reflects the changing relationship of the central pixels at different moments, which provides an adequate clue for the description of temporal intensity.

**Figure 6:**
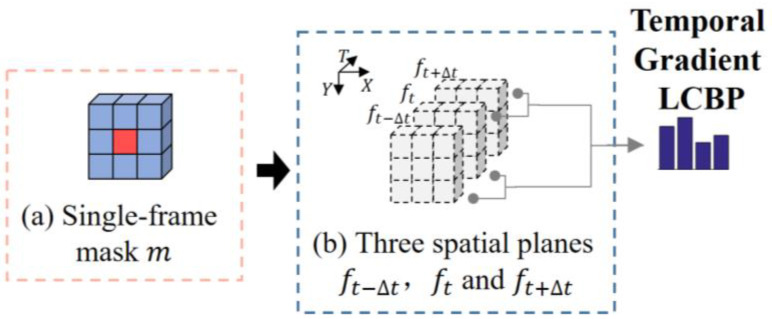
The encoding process of Temporal Gradient LCBP. (**a**) The diagram of single-frame mask. (**b**) The sampling range of the spatial planes $f_{t-\Delta t}$, $f_{t}$ and $f_{t+\Delta t}$.

Specifically, as shown in Figure 6a, the blue region in the single-frame template m is assigned a value of −1, and the red region, which is the single-frame center pixel of the cube, is assigned as 8. The local spatial response values of the different frames $S_{i}$ are obtained by doing convolutional operations on the planes $f_{t-\Delta t}$, $f_{t}$ and $f_{t+\Delta t}$ of the different frames with the single-frame mask m. The $S_{i}$ can be calculated as follows:

(7) $$S_{i}=m*f_{i},$$

where, ∗ denotes the convolutional operation. The temporal gradient $Grad\left(S_{i+\Delta t,},\;S_{i}\right)$ between two adjacent frames can be defined as:

(8) $$Grad\left(S_{i+\Delta t,},S_{i}\right)=\left(S_{i+\Delta t}-S_{i}\right)/\Delta t,$$

Temporal Gradient LCBP defines the gradient changes by the following four cases:

(1) the binary code is 11 when $Grad\left(S_{i+\Delta t,},S_{i}\right)$ ≥ 0 and $Grad\left(S_{i,},S_{i-\Delta t}\right)$ ≥ 0, indicating that the pixel brightness of the three frames is gradually brightened;
(2) the binary code is 00 when $Grad\left(S_{i+\Delta t,},S_{i}\right)$ < 0 and $Grad\left(S_{i,},S_{i-\Delta t}\right)$ < 0, indicating that the brightness of the three frames is gradually darkened;
(3) the binary code is 10 when $Grad\left(S_{i+\Delta t,},S_{i}\right)$ < 0 and $Grad\left(S_{i,},S_{i-\Delta t}\right)$ ≥ 0, indicating that the brightness of the three frames is brightened and then darkened;
(4) the binary code is 11 when $Grad\left(S_{i+\Delta t,},S_{i}\right)$ ≥ 0 and $Grad\left(S_{i,},S_{i-\Delta t}\right)$ < 0, indicating that the brightness of the three frames is darkened and then brightened.

In all four cases, the intensity changes in the temporal direction are judged by two inter-frame gradient variation, so that the sampling range covers all pixels in the cube space. The first two cases reveal that the local region contains continuously displaced motions along the same direction, usually indicating a significant change in the brightness of the pixel. By contrast, the latter two cases imply that the temporal direction of the local region displacement is unstable, usually indicating that the brightness variation of the pixel is fluctuating. The feature histogram is calculated based on the four cases as a Temporal Gradient LCBP feature, which constitutes a complete Enhanced LCBP feature with the other two binary features. The more compact and discriminative representation is further obtained using the Multi-Regional Joint Sparse Learning model while ensuring that time and space domain variations are encoded.

### 3.2. Multi-Regional Joint Sparse Learning

For the LBP-based ME recognition method, MEs are predicted by the feature of the local areas extracted from the descriptor. Since the statistical features used in the LBP-based methods do not contain local location information, dividing the image into grids is the solution to extract the location information.

In fact, it is clear that not all grids contain valid recognition features for predicting MEs. Some of these grid features are redundant, which reduces the efficiency of the LBP-based classification scheme. The facial action units account for the creation of such redundant features. Specifically, the Facial Action Coding System (FACS) define basic facial movement units based on the dynamic characteristics of particular facial muscles, and the MEs can be decomposed into individual action units, which are the typical features that are used to distinguish different kinds of emotions. Thus, for different local regions, the Multi-Regional Joint Sparse Learning is proposed to quantify their various contributions to the recognition task.

#### 3.2.1. Construct Objective Function

As mentioned earlier, we hope to select those facial regions that trigger MEs, since only these facial grids contribute significantly to the recognition task. To address this issue, the linear regression regularization is an effective solution to perform Multi-Regional Joint Sparse Learning. Regularization is an implementation of the structural risk minimization strategy, which selects models with both less empirical risk and model complexity by adding a regularization term to the empirical risk. Furthermore, the global feature selection is formulated as a multi-task regression problem by treating the feature selection for each facial grid as a single regression task. Consider a multi-task regression problem with 𝛽 facial grids, an efficient approach is to estimate the feature selection weights W by minimizing the following objective function:

(9) $$\underset{W}{\min}\sum_{i=1}^{\beta }\|X_{i}w_{i}-Y_{i}\|{}_{F}^{2}+\theta_{1}{\left\|W\right\|}_{F}^{2}$$

where $X_{i}={\left[x_{1},x_{2},\ldots ,x_{s}\right]}^{T}$ denotes the data matrix consisting of s training samples of the $i^{th}$ facial grid, and $Y_{i}={\left[y_{1},y_{2},\ldots ,y_{s}\right]}^{T}$ denotes the label matrix consisting of s corresponding targets of the $i^{th}$ facial grid. $w_{i}$ indicates the regression coefficient vector of the $i^{th}$ facial grid, and all the coefficient vectors of the facial grid are from a coefficient matrix $W=\left[w_{1},\ldots ,w_{i},\ldots ,w_{\beta }\right]$. The first term in Equation (9) calculates the empirical loss and the second controls for the generalization error. $\theta_{1}$ is the regularization factor that controls the relative weights of the two terms. ${\left\|.\right\|}_{F}$ is the Frobenius norm of a matrix.

Further, since the variation in appearance between different classes is not evident, this negatively affects the discriminative power of the representations. In order to enhance the inter-class differences of the representation, the inter-class distance penalty term is introduced to constrain the feature distance among the different classes, which allows the inter-class distance to be improved. Thus, we add the constraint of interclass distance to the proposed Multi-Regional Joint Sparse Learning model.

Moreover, the limited training sample results in a greater feature dimension compared with the sample size. In response, dimensionality reduction is an effective strategy to address the risk of over-fitting. Least Absolute Shrinkage and Selection Operator (LASSO) is a regression analysis method that performs both feature selection and regularization. It can be used for parameter selection through parameter reduction; thus, the purpose of dimensionality reduction can be achieved. Although LASSO can produce some regression coefficients strictly equal to 0 to obtain models with strong explanatory power, it needs to choose several single variables instead of general factor selection, to represent the pixels of each image [31]. For this issue, the proposed model adopts Group LASSO regularized feature selection based on $\ell_{2,1}$-norm penalty, which solves the feature selection problem of multi-regional grouping variables. Specifically, the selection and classification of features for each image grid can be considered as a single task, and thus it can be formulated as a multi-task regression problem for all grids. Group LASSO imposes group constraint on the data based on sparse representation, which encourages the majority of feature weighting coefficients to be zero. A few feature-weighting coefficients are non-zero as the shared features for all tasks, ensuring that the same features are selected between different image grids. Ultimately, the optimal solution assigns relatively large weights to features, which provide classification information, and zero or smaller weights vice versa. These features with non-zero weights are used for classification as optimized compact representations. Thus, we can define the feature selection objective function for Multi-Regional Joint Sparse Learning as follows:

(10) $$\underset{W}{\min}\sum_{i=1}^{\beta }\|X_{i}w_{i}-Y_{i}\|{}_{F}^{2}+\theta_{1}{\left\|W\right\|}_{F}^{2}+\delta \sum_{u=1}^{c}\sum_{v=1}^{c}{\left(m_{u}-m_{v}\right)}^{-2}+\theta_{2}{\left\|W\right\|}_{2,1}$$

The third term in Equation (10) is the constraint of interclass distance. δ≥0 is the penalty term parameter used to control the constraint, $m_{u}$ represents the mean of weighted feature for all samples belonging to class u, which is the class center in the feature space for all samples of class u, and u ≥ v. c represents the total number of classes in the training sample. The discriminative power of weighted representations is enhanced by increasing the distance constraint between different classes. The last term is the $\ell_{2,1}$-norm. $\theta_{2}$ is the regularization coefficient, and ${\left\|W\right\|}_{2,1}=\overset{r}{\underset{i=1}{\sum }}{\|w}^{i}\|{}_{2}$ where $w_{i}$ denotes the $i^{th}$ row of matrix W, and r is the number of rows. This leads to the exploitation of relevant information between different groups, resulting in a similar sparse pattern among the variables between the groups.

#### 3.2.2. Optimization

Since the proposed objective function contains the $\ell_{2,1}$-norm, it causes the objective function to be non-differentiable and non-smooth. Since the objective function can be considered as a combination of smooth and non-smooth terms, the Accelerated Proximal Gradient (APG) algorithm [32] can be used to solve this group sparsity problem. First, the objective function can consist of the smooth term f(W) and the non-smooth term g(W).

(11) $$f\left(W\right)=\underset{W}{\min}\sum_{i=1}^{B}\|X_{i}w_{i}-Y_{i}\|{}_{F}^{2}+\theta_{1}{\left\|W\right\|}_{F}^{2}+\delta \sum_{u=1}^{c}\sum_{v=1}^{c}{\left(m_{u}-m_{v}\right)}^{-2},$$

(12) $$g\left(W\right)=\theta_{2}{\left\|W\right\|}_{2,1},$$

Second, the function $H_{\rho }\left(W,W^{\left(k\right)}\right)$ is used to approximate f(W) +  g(W):

(13) $$H_{\rho }\left(W,W^{\left(k\right)}\right)=f\left(W^{\left(k\right)}\right)+W-W^{\left(k\right)},\nabla f\left(W^{\left(k\right)}\right)+\frac{1}{2}\|W-W^{\left(k\right)}\|{}_{F}^{2}+g\left(W\right),$$

where ${\left\|.\right\|}_{F}$ is the Frobenius norm of a matrix. $\nabla f\left(W^{\left(k\right)}\right)$ is the gradient of f(W) at k iterations of the point $W^{\left(k\right)}$. Moreover, ρ is the step length, which can be obtained by a linear search. The update steps of the APG algorithm can be calculated as follows:

(14) $$W^{\left(k+1\right)}=arg\;\underset{W}{\min}\|W-E^{\left(k\right)}\|{}_{F}^{2}+\frac{1}{\rho }g\left(W\right),$$

and $E^{\left(k\right)}=W^{\left(k\right)}-\frac{1}{\rho }\nabla f\left(W^{\left(k\right)}\right)$. Furthermore, according to the method proposed in [32], $H_{\rho }\left(W,W^{\left(k\right)}\right)$ can be calculated by the following equation:

(15) $$Q^{\left(k+1\right)}=W^{\left(k+1\right)}+\frac{\left(1-\varphi_{k}\right)\varphi_{k+1}}{\varphi_{k}}\left(W^{\left(k+1\right)}-W^{\left(k\right)}\right),$$

where $\varphi_{k}=\frac{2}{k+2}$. The convergence speed of this algorithm can reach $O\left(\frac{1}{K^{2}}\right)$, and K is the maximum number of iterations.

### 3.3. Multi-Kernel Support Vector Machine

A multi-kernel SVM [33] can effectively integrate different training features. To be specific, different features are mapped to their respective feature spaces using independent kernel functions, and the combinations of multiple feature spaces can fully exploit the different feature mapping capabilities of each kernel. The spatial and temporal domain features obtained by optimizing the objective function are fed into the multi-kernel SVM, which can be mapped to a higher-dimensional feature space, respectively. The employed kernel functions are linear kernels, which can be expressed as:

(16) $$\kappa_{z}\left(x_{i}^{z},x_{j}^{z}\right)=\gamma {\left(x_{i}^{z}\right)}^{T}\gamma \left(x_{j}^{z}\right),$$

where $\kappa_{z}$ is the kernel function for the two training samples on the zth feature. γ(x) is used to represent the kernel-induced mapping function. Further, the integrated multi-kernel function of the spatial and temporal features can be represented as:

(17) $$\kappa_{z}(x_{i},x_{j})=\sum_{z=1}^{2}c_{z}\kappa_{z}(x_{i}^{z},x_{j}^{z}),$$

The dual form of a multi-kernel SVM can be represented as:

(18) $$\underset{\alpha }{\max}\sum_{i=1}^{N}\alpha_{i}-\frac{1}{2}\sum_{i,j=1}^{N}\alpha_{i}\alpha_{j}y_{i}y_{j}\sum_{z=1}^{2}c_{z}\kappa_{z}(x_{i}^{z},x_{j}^{z}),$$

where $\alpha_{i}$ is the Lagrangian multiplier corresponding to sample i. N indicates the number of training samples. Herein, we used the LIBSVM toolkit [34] to implement the SVM classifier and the parameters $c_{z}$ for each feature matrix are obtained by cross-validation in training.

## 4. Experiments and Analysis

### 4.1. Datasets

To verify the effectiveness of the proposed method, the proposed algorithm will be evaluated in four spontaneous ME databases, including Spontaneous Micro-Expression Database (SMIC) [35], Chinese Academy of Sciences Micro-expression (CASME-I) [36], Chinese Academy of Sciences Micro-expression2 (CASME-II) [37], and Spontaneous Micro-facial Movement Dataset (SAMM) [38]. Table 1 shows the detailed properties of the four databases.

SMIC contains 164 ME videos across 16 subjects. All samples are classified into three categories, i.e., Positive (51 samples), Negative (70 samples), and Surprising (43 samples). SMIC data are available in three versions: high-speed camera (HS) version at 100 fps, normal vision camera (VIS) version at 25 fps, and near-infrared camera (NIR) version at 25 fps. In this paper, the HS samples are used for experiments.

CASME-I contains 195 ME video clips from 19 subjects. All samples are coded with the onset frames, apex frames and offset frames, and marked with action units (AUs) and expression states. There are eight classes of MEs in this database, i.e., Tense, Disgust, Repression, Surprise, Happiness, Fear, Sadness, and Contempt. Due to the limited sample size in the categories of Happiness, Fear, Contempt, and Sadness, we chose the remaining four categories: Tension (69 samples), Disgust (44 samples), Repression (38 samples), and Surprise (20 samples) for the experiment.

CASME-II is an updated version of CASME-I and contains 247 ME videos from 26 subjects. All samples were classified into seven classes, i.e., Disgust, Repression, Surprise, Happiness, Others, Fear, and Sadness, and all samples were also marked with onset frame, apex frame, offset frame, and action units (AUs). The temporal resolution of CASME-II was improved from 60 fps in CASME-I to 200 fps. The spatial resolution has also improved, with a partial face resolution of 280 × 340. Because of the limited sample size in the Fear and Sadness groups, the remaining five classes were selected for the experiment, i.e., Disgust (64 samples), Repression (27 samples), Surprise (25 samples), Happiness (32 samples), and Others (99 samples).

SAMM database contains 159 ME video clips from 29 subjects. All samples were recorded at a time resolution of 200 frames per second. These samples were divided into seven AU based objective classes. For the convenience of the comparison with our previous research, the samples used for the experiment were divided into five classes based on the emotional link of AUs in FACS, which consisted of Happiness (24 samples), Surprise (13 samples), Anger (20 samples), Disgust (8 samples), and Sadness (3 samples).

### 4.2. Implementation Details

For the convenience in the analysis of the results, our implementation follows the same data pre-processing and evaluation protocol as in the previous work:

(1) pre-cropped video frames provided in SMIC, CASME, and CASME-II were used as experimental data. For images in SAMM, facial images are extracted using the Dlib face detection [39] and resized to a uniform image resolution of 128 × 128;
(2) due to the different time lengths of these four datasets, the temporal interpolation model (TIM) [40] is used to fix the time length of the image sequence to 20;
(3) leave-one-subject-out (LOSO) cross-validation [41] is used to evaluate the performance of the model. Specifically, all samples from a subject are used as a test set, and the rest are used for training. LOSO cross-validation ensures that the same subject samples are not duplicated in the training and test sets, improving sample-efficiency while ensuring the reliability of results;
(4) overall recognition accuracy and F1 scores are used to evaluate the performance of the model. Recognition accuracy can be calculated by the following equation:
  (19) $$Accuracy=\frac{\sum_{i=1}^{N}T_{i}}{\sum_{i=1}^{N}S_{i}}\times 100\%,$$
  where $T_{i}$ indicates the number of correct predictions of each subject, $S_{i}$ represents the total number of samples for each subject, and N denotes the number of subjects. In addition, the F1 score is used to reveal the classifier’s recognition performance for each category due to the imbalance of sample distribution in the MEs dataset. The F1 score can be calculated as:
  (20) $$F=\frac{1}{C}\sum_{i=1}^{C}\frac{2p_{i}\times r_{i}}{p_{i}+r_{i}},$$
  where $p_{i}$ and $r_{i}$ are the precision and recall of the $i^{th}$ ME class, respectively, and c is the number of MEs classes.

### 4.3. Parameter Evaluation

The binary feature histogram of Enhanced LCBP consists of three complementary spatial and temporal descriptors, i.e., Spatial Difference LCBP, Temporal Direction LCBP, Temporal Gradient LCBP. Table 2 presents the experimental results of different combinations of the spatial and temporal descriptors on CASME-II. The results are obtained using 8 × 8 grid division parameters. The experimental model of combining different descriptors do not contain the Multi-Regional Joint Sparse Learning and are implemented using the SVM classifier. Spatial Difference LCBP is mainly responsible for capturing the spatial features in the cube space, while describing the spatial difference between different frames. In the single descriptor experiment, the recognition rate of using Spatial Difference LCBP is only 56.09%, which is due to the fact that the key to the ME recognition task is to extract temporal variation. The Spatial Difference LCBP is employed as an auxiliary binary descriptor to help Enhanced LCBP enrich the spatial representation. As the time-domain feature descriptor, Temporal Direction LCBP shows better recognition results in the single descriptor experiment. The accuracy is 5.7% higher than using Spatial Difference LCBP alone. Besides, the results reveal that the combination of Temporal Direction LCBP and Temporal Gradient LCBP can effectively integrate the motion direction with the motion tendency, with an improvement of 4.88% compared with a single Temporal Direction LCBP descriptor. This improvement evidences that the combined result of Temporal Direction LCBP and Temporal Gradient LCBP are enhanced for time-domain feature extraction. Moreover, the recognition rate obtained by the combination of Temporal Direction LCBP and Spatial Difference LCBP is not significant compared with that obtained by a single Temporal Direction LCBP descriptor. However, the combined results of the Temporal Direction LCBP and Spatial Difference LCBP are not significantly improved. It shows that the ability to capture time-domain features has a significant impact on the performance of the model. The recognition rate reaches 69.10% with the combination of three descriptors fusion and an SVM classifier. Such a result is consistent with our assumption that fusing spatiotemporal features provides a more detailed representation of subtle changes. The results for all three descriptor combinations are significantly improved compared with a single descriptor or any combination of the two.

**Figure 7:**
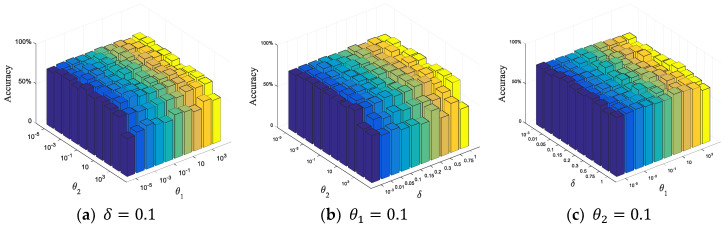
The histogram of the recognition accuracy with respect to the three parameters; (**a**–**c**) show the recognition accuracy obtained by adjusting the remaining two parameters when the corresponding parameter is fixed at 0.1, respectively. The X and Y axes represent the values of different parameters, and the z-axis represents the classification accuracy of ME recognition.

In addition, there are three parameters that need to be adjusted in the objective function of our proposed Multi-Regional Joint Sparse Learning, i.e., $\theta_{1}$, $\theta_{2}$ and δ. This is because these three parameters balance the relative contributions of the group sparse regular term and the constraint term of interclass distance. Therefore, the effect of different parameter settings on classification performance needs to be studied in terms of parameter selection. Specifically, $\theta_{1}$ and $\theta_{2}$ vary in the range of $\left\{10^{-5},\cdots ,10^{-1},\cdots ,10^{5}\right\}$ and δ in the range of {0.001, 0.01, 0.05, 0.1, 0.2⋯,1}. The parameter setting strategy is to adjust the remaining two parameters by first fixing the value to one parameter. First, the value of δ is fixed to 0.1, and we change $\theta_{1}$ and $\theta_{2}$ within their respective adjustable ranges. Then we fix $\theta_{1}$ to 0.1 and change $\theta_{2}$ and δ within their respective adjustable ranges. Finally, we fix the value of $\theta_{2}$ to 0.1 and change the remaining two parameters in the corresponding adjustable ranges. The experiments are implemented based on CASME-II, and the grid division parameter is set to 8 × 8. The classifier employs the multi-kernel SVM. The histogram of the recognition accuracy with the variation of parameters is shown in Figure 7. The results show slight fluctuations when the proposed method fixes one parameter and adjusts the remaining two parameters, indicating that our method is not sensitive to the variation in parameter values.

Furthermore, since grid division parameters affect model performance, fewer grids result in insufficient location information and detailed changes, while excessive grids may bring the noise. Therefore, we discuss the impact of different grid division parameters on recognition performance. The experimental result is shown in Table 3. The optimal results in CASME-I and CASME-II are obtained using the 8 × 8 grid division parameters. The best results in SMIC and SAMM databases are obtained using 6 × 6 grid division parameters. It can be seen that the differences in the results among different grid delineation parameters are within acceptable ranges.

### 4.4. Comparison with Other State-of-the-Art Methods

**Figure 8:**
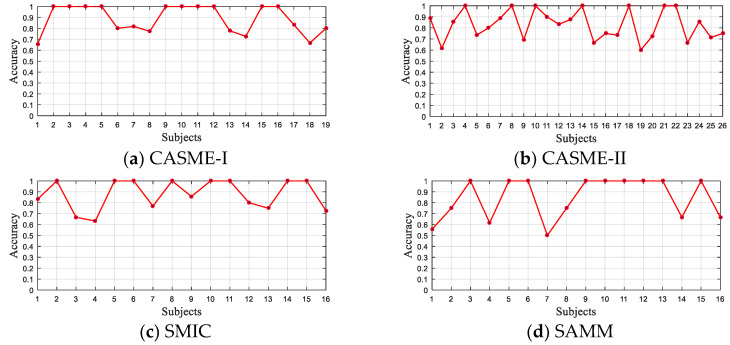
The accuracy of each subject for the predictions on CASME-I, CASME-II, SMIC, and SAMM, respectively.

To assess the performance of the proposed method, we compared the results with other mainstream approaches based on four published datasets. Figure 8 illustrates the LOSO recognition results obtained by our method in four datasets. The horizontal coordinates in the figure represent the different subjects, and the vertical coordinates represent the recognition rate obtained by each subject. The results show that half of the subjects in CASME-I, SAMM, and SMIC achieved 100% recognition rate. Although the number of subjects that achieved 100% accuracy rate in CASME-II is less than half the total, the prediction accuracy rate for all subjects exceeded 60%. Meanwhile, due to the small sample size of some testing sets in SAMM, this resulted in subject 7 recognizing one correct sample and thus achieving the 50% accuracy rate. By further calculating the average accuracy of the LOSO results obtained for each dataset, it can be concluded that CASME-I: 88.70%, CASME-II: 82.90%, SMIC: 87.72%, and SAMM: 88.12%. It can be seen that the average LOSO recognition rate of the four datasets exceeded 80%, indicating the good reliability and accuracy of our method.

**Figure 9:**
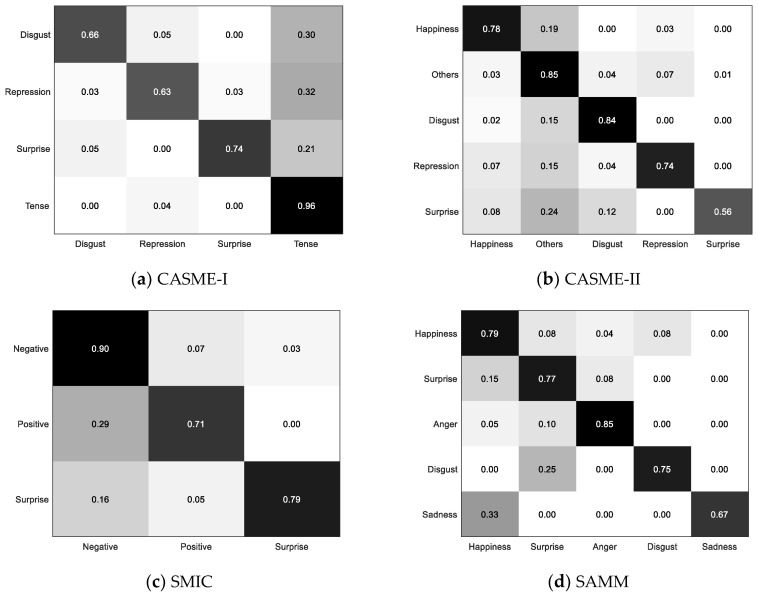
The confusion matrix is calculated based on the LOSO results of CASME-I, CASME-II, SMIC, and SAMM, respectively.

Based on the LOSO results obtained by the proposed method, the confusion matrix is calculated to assess the model’s recognition performance for each category. As shown in Figure 9, the proposed method has a higher prediction accuracy for the Negative in SMIC compared with the others. The same situation occurs with the Tense in CASME-I and Others in CASME-II. This is owing to the fact that ME recognition datasets all suffer from unbalanced class distribution, and these expression categories with larger sample sizes are more likely to receive attention from classification algorithms. It can be seen that SMIC has the smallest accuracy difference among categories, which is attributed to the fact that the unbalanced class distribution is more severe in all three remaining databases. The unbalanced class distribution poses significant challenges to the classification algorithm. However, the results show that the performance gaps of different categories are mitigated compared with our previous study [21]. The discriminability of weighted features is enhanced by the term of interclass distance constraint in the objective function, which helps to reduce the interclass accuracy gap. Our model guarantees a recognition rate of more than half for different categories. The difference in accuracy among the categories is within an acceptable range.

Finally, we compare the best results obtained by our method with the baseline method and the other representative algorithms. Since the performance of different protocols varies substantially, we only compare methods that use the same LOSO strategy. The baseline methods include LBP-TOP [18] and LBP-SIP [20], which use the same grid division parameters as our method. The time-domain radius of LBP-TOP is set to 2, and the number of sampling points is set to 8. The asterisk (*) indicates that we are using the experimental results reported from corresponding literature. The results of the comparison are shown in Table 4. The proposed method achieves better recognition rates in four datasets than the other algorithms. Our method achieved 78.23% and 78.45% recognition accuracy in the CASME-I and CASME-II, and 79.26% and 79.41% recognition rates in the SMIC and SAMM, respectively. Compared with the baseline approach, the proposed method shows superior performance. Compared with the optical-flow-based methods Sparse MDMO [15] and Bi-WOOF+Phase [42], our method exhibits an accuracy improvement of 11.5% and 15.9% in CASME-II, respectively. The proposed method improved the accuracy of CASME-II by 4.51% and 8.35%, respectively, compared with the results reported by the recent handcrafted methods ELBPTOP [43] and LCBP [21]. This advantage is facilitated by the fact that the Enhanced LCBP consisted of spatial and temporal descriptors focused on capturing spatiotemporal information. Moreover, the combination of Multi-Regional Joint Sparse Learning allows the use of relevant information between different regions, which helps select more discriminating features.

Table 5 compares the F1-scores of our method, LBP-TOP, and LCBP in all four datasets. The results show that our method is better than the other two methods in four aspects. Compared with LCBP, the proposed method improved F1 scores in SMIC and CASME-II by 0.1162 and 0.0547, respectively, proving that the improvement of the proposed method is effective compared with our previous study. Table 6 summarizes our approach in comparison to the deep learning approach in the CASME-II and SMIC. In contrast to the deep learning approach, our method still shows an advantage in terms of recognition rate and F1 score. It can be demonstrated through the above comparative experiments that our method is valid for the ME recognition task.

## 5. Conclusions

In this paper, a sparse binary descriptor for ME recognition based on the Enhanced LCBP is proposed. In comparison to other binary descriptors, the proposed Enhanced LCBP contains the complementary spatial and temporal binary features that allows a greater focus on capturing spatial differences and temporal directions. Moreover, Multi-Regional Joint Sparse Learning is proposed to measure the contribution of different regions, guaranteeing a similar sparse pattern between features of different image grids. Moreover, the interclass distance constraint allows the proposed method to capture more discriminating binary representations. The effect of the Enhanced LCBP descriptor and Multi-Regional Joint Sparse Learning on recognition performance is quantitatively evaluated through parameter experiments. The results show that the combination of Enhanced LCBP and Multi-Regional Joint Sparse Learning can effectively improve the performance of the model. The comparison results with mainstream methods on four datasets show that our approach achieves promising results. This work will contribute to the further exploration of sparse binary descriptors, which will improve the prediction of MEs, and thus combining the advantages of handcrafted features with deep learning technology will be our future work.

## Figures and Tables

**Table 1:** Detailed attributes of micro-expression (ME) datasets.

Datasets	Number of Samples	Frame Rate (fps)	Resolution	Action Units Labels
Spontaneous Micro-Expression Database (SMIC)	164	100	640 × 480	×
Chinese Academy of Sciences Micro-expression (CASME-I)	195	60	Part A: 1280 × 720	√
			Part B: 640 × 480	
Chinese Academy of Sciences Micro-expression2 (CAMSE-II)	247	200	640 × 480	√
Spontaneous Micro-facial Movement Dataset (SAMM)	159	200	2040 × 1088	√

**Table 2:** Performance comparison between different combinations of spatial and temporal descriptors on CASME-II. Experiments are conducted without Multi-Regional Joint Sparse Learning.

Different Combinations of Descriptors	Recognition Rate (%)
Spatial Difference LCBP	56.09%
Temporal Direction LCBP	61.79%
Temporal Gradient LCBP + Temporal Direction LCBP	66.67%
Spatial Difference LCBP + Temporal Direction LCBP	64.23%
Three Descriptors	69.10%

**Table 3:** Experimental results of the different grid division parameters on CASME-I, CASME-II, SMIC, and SAMM.

Grid Division Parameters	CASME-I	CASME-II	SMIC	SAMM
5 × 5	75.29%	75.20%	77.44%	77.20%
6 × 6	76.47%	76.82%	79.26%	79.41%
7 × 7	77.06%	77.64%	78.44%	78.69%
8 × 8	78.23%	78.45%	78.05%	78.43%
9 × 9	76.76%	76.42%	77.74%	76.47%

**Table 4:** Comparison of recognition results among the proposed micro-expression (ME) recognition method and recent state-of-the-art methods.

Method	SMIC	CASME-I	CASME-II	SAMM
LBP-TOP [18]	42.36%	42.83%	40.49%	41.83%
LBP-SIP [20]	44.31%	43.56%	42.07%	42.72%
FDM * [14]	54.88%	56.14%	45.93%	N/A
MDMO * [13]	58.97%	56.29%	51.69%	N/A
Sparse MDMO * [15]	70.51%	74.83%	66.95%	N/A
HIGO * [8]	68.29%	N/A	67.21%	N/A
STLBP-IP * [22]	57.93%	N/A	59.51%	N/A
DISTLBP-RIP * [23]	63.41%	64.33%	64.78%	N/A
LCBP * [21]	71.30%	76.36%	70.10%	76.10%
ELBPTOP * [43]	69.06%	N/A	73.94%	63.44%
Bi-WOOF+Phase * [42]	68.29%	N/A	62.55%	N/A
Our Method	79.26%	78.23%	78.45%	79.41%

* The experimental results presented here are obtained from the corresponding literature.

**Table 5:** The comparison of F1-scores on four spontaneous MEs databases.

Method	SMIC	CASME-I	CASME-II	SAMM
LBP-TOP [18]	0.3740	0.3681	0.3475	0.3601
LCBP [21]	0.6861	0.6595	0.7033	0.7543
Our Method	0.8023	0.7542	0.7580	0.7900

**Table 6:** Comparison of recognition results between the proposed method and deep learning methods on CASME-II and SMIC.

Method	SMIC		CASME-II	
	Accuracy	F1-Score	Accuracy	F1-Score
ELRCN * [24]	N/A	N/A	52.44%	0.5000
3D flow-based CNN * [26]	55.49%	N/A	59.11%	N/A
TSCNN * [28]	72.74%	0.7236	74.05%	0.7327
SSSN * [44]	63.41%	0.6329	71.19%	0.7151
DSSN * [44]	63.41%	0.6462	70.78%	0.7297
Our Method	79.26%	0.8023	78.45%	0.7580

* The experimental results presented here are obtained from the corresponding literature.

## Abbreviations

LBP-TOP [18]	Local Binary Patterns on Three Orthogonal Planes
LBP-SIP [20]	Local Binary Pattern with Six Intersection Points
FDM * [14]	Facial Dynamics Map
MDMO * [13]	Main Directional Mean Optical-flow
Sparse MDMO * [15]	Sparse Main Directional Mean Optical-flow
HIGO * [8]	Histogram of Image Gradient Orientation
STLBP-IP * [22]	Spatiotemporal Local Binary Pattern with an Integral Projection
DISTLBP-RIP * [23]	Discriminative Spatiotemporal Local Binary Pattern with Revisited Integral Projection
LCBP * [21]	Local Cube Binary Patterns
ELBPTOP * [42]	Extended Local Binary Patterns on Three Orthogonal Planes
Bi-WOOF+Phase * [43]	Bi-Weighted Oriented Optical Flow with phase information
ELRCN * [24]	Enriched Long-term Recurrent Convolutional Network
3D flow-based CNN * [26]	3D flow-based convolutional neural networks
TSCNN * [28]	Transferring Long-term Convolutional Neural Network
SSSN * [44]	Single-Stream Shallow Network
DSSN * [44]	Dual-Stream Shallow Network
